# *FANCA* Gene Mutations with 8 Novel Molecular Changes in Indian Fanconi Anemia Patients

**DOI:** 10.1371/journal.pone.0147016

**Published:** 2016-01-22

**Authors:** Avani Solanki, Purvi Mohanty, Pallavi Shukla, Anita Rao, Kanjaksha Ghosh, Babu Rao Vundinti

**Affiliations:** Department of Cytogenetics, National Institute of Immunohaematology (ICMR), Mumbai, Maharashtra, India; Oregon Health and Science University, UNITED STATES

## Abstract

Fanconi anemia (FA), a rare heterogeneous genetic disorder, is known to be associated with 19 genes and a spectrum of clinical features. We studied *FANCA* molecular changes in 34 unrelated and 2 siblings of Indian patients with FA and have identified 26 different molecular changes of *FANCA* gene, of which 8 were novel mutations (a small deletion c.2500delC, 4 non-sense mutations c.2182C>T, c.2630C>G, c.3677C>G, c.3189G>A; and 3 missense mutations; c.1273G>C, c.3679 G>C, and c.3992 T>C). Among these only 16 patients could be assigned FA-A complementation group, because we could not confirm single exon deletions detected by MLPA or cDNA amplification by secondary confirmation method and due to presence of heterozygous non-pathogenic variations or heterozygous pathogenic mutations. An effective molecular screening strategy should be developed for confirmation of these mutations and determining the breakpoints for single exon deletions.

## Introduction

Fanconi Anemia (FA; MIM no. 227650) is a rare autosomal recessive or X-linked genomic instability disorder affecting all ethnic groups with an incidence of 1 in 350,000 live births [1, 2]. Currently, FA is the most common inherited cause of bone marrow failure, characterized by numerous congenital anomalies (~60%), cellular hypersensitivity to DNA inter-strand crosslinking (ICL) agents such as Mitomycin C (MMC) and Diepoxybutane (DEB) and increased predisposition to both haematological and solid tumors [1, 2].The clinical diagnosis of FA is often complicated by heterogeneity in phenotype of FA patients [3]. The diagnostic criterion for FA therefore, relies on the hypersensitivity of cells cultured from FA patients to DNA cross-linking agents such as MMC and DEB. However, molecular analysis is still required for characterization of FA patients and demonstrating pathogenic mutation in FA genes. So far, 19 different FA causing genes (*FANCA*-*T*) forming a part of FA pathway have been identified in FA which has led to a better understanding of both the phenotype and genotype of the disease condition [4]. Among all *FANCA*, *FANCC*, *and FANCG* complementation groups account for the disease in 60%, 15%, and 10% of FA families respectively, whereas mutations in the other genes occur less frequently (0.1%-4%) [5].The proteins encoded by these genes participate in pathways that are involved in detection and resolution of DNA interstrand-crosslinks, maintenance of hematopoietic stem cells and prevention of tumorigenesis [6].The challenge of understanding the etiology of this complex disease needs to be addressed through comprehensive genotype-phenotype correlation studies and the wide screening of multiple genes involved. We investigated the frequency, type and clinical impact of the most common FA complementation group FA-A in Indian population.

## Material and Methods

### Patients

The study was carried out on 550 patients suspected with Fanconi anemia. Patients were referred from all over India. The patients included were children or adults with classical clinical presentation of FA (like Bone marrow failure, skin pigmentation abnormalities, and congenital anomalies) and suspected FA patients without a full clinical presentation (only congenital anomalies and/or skin pigmentation abnormalities without onset of hematological changes). The age of these patients ranged from 1 year to 30 years. The study protocols were approved by Institutional Ethics Committee for research on Human subjects of National Institute of Immunohematology (ICMR). In case of children, the written informed consent was obtained from parents. The clinical details including age, sex, parental consanguinity, parental age, reproductive history and haematological profile were recorded in an intricately designed proforma. The peripheral blood samples were collected in heparin (7cc) and EDTA (4cc) vacutainers from the subjects with the written consent.

### Chromosomal breakage test

Chromosome breakage study was done using Phytohemagglutinin (PHA) stimulated lymphocyte cultures induced with Mitomycin C (MMC) with final concentration of 40ng/mL and incubated at 37°C for 72 hrs. Cells were arrested with colchicines at 68^th^ hour at metaphase stage, followed by hypotonic solution treatment with 0.075M potassium chloride, and cells were fixed with Carnoy’s fixative (3:1 methanol: glacial acetic acid). The cells were dropped on pre-chilled slides and stained with Giemsa stain. A total of fifty metaphases were scored under bright field microscope and chromosomal breakages and radial forms were recorded and compared negative control (or non-FA) sample each time [7].

### FANCD2 monoubiquitination detection by Western blot

Peripheral blood mononuclear cells were isolated using 5 mL of heparinized blood from patients by centrifugation over Histopaque 1077 (Sigma). The cells were washed twice and cultured in RPMI 1640 containing 10% heat inactivated fetal bovine serum (Sigma) and 2mM glutamine, penicillin and streptomycin. Cultures were stimulated with PHA for 72 hrs. The cells were induced with or without DNA inter-strand cross-linking agent MMC (40 ng/mL), after 24hrsof incubation. Cell cultures were maintained in a humidified incubator containing 5% CO_2_ at 37°C and the cell lysate was prepared after 72 hours of culture. Lysis buffer consisting of Tris.HCl, SDS, and BME was used. The pellet of cultured cells was lysed in the lysis buffer on ice for 30 minutes. Lysate was heated for 5 minutes at 96°C and loaded on 3–8% gradient tris–acetate gel for electrophoresis. Proteins were transferred to nitrocellulose using iBlot® Dry Blotting System (Invitrogen). The nitrocellulose membrane was blocked with 5% non-fat dried milk in TBS-T (50mM Tris–HCl, 150mM NaCl, 0.1% Tween20) and incubated overnight with primary anti-FANCD2 mouse monoclonal antibody diluted 1:300 followed by incubation with the secondary antibody linked to horseradish peroxidase. Detection was done using ECL PLUS kit (GE Healthcare). The short or small band (S) of 155 kDa refers to the non-ubiquitinated protein and the long or large (L) band of 162 kDa refers to the monoubiquitinated FANCD2 protein.

### Mutation Screening of *FANCA* gene

#### Genomic DNA and total RNA extraction from peripheral blood

The total genomic DNA and RNA was extracted from EDTA peripheral blood using QIAamp DNA Blood Mini Kit(Qiagen) and QIAamp RNA Blood Mini Kit (Qiagen) according to the manufacturer's instructions. The concentrations of DNA and RNA were determined on nanodrop spectrophotometer. The RNA reverse transcribed to first-strand cDNA by using RevertAid H minus First Strand cDNA Synthesis Kit (Thermo Scientific).

#### Screening for large deletions

Multiplex Ligation-dependent Probe Amplification (MLPA) was done using SALSA MLPA kits (P031 & P032 FANCA) for *FANCA* gene. Briefly, target DNA was denatured for 5 minutes at 98°C, probe mix was added, after which the mixture was heated for 1 minute at 98°C and incubated at 60°C overnight (16 hr); after addition of ligase, the mixture was incubated at 54°C for 15 minutes. Ligase was subsequently inactivated at 98°C for 5 minutes. Next, ligation product was transferred to PCR mix. The PCR reaction was carried out for 35 cycles (30 seconds at 95°C, 30 seconds at 60°C, and 60 seconds at 72°C). The fragments were analyzed on an ABI model 3130 capillary sequencer (Applied Biosystems) using genescan-TAMRA 500 size standard (Applied Biosystems).

#### PCR amplification of the cDNA by RT-PCR and Direct Sequencing

Six pairs of primers were designed according to the *FANCA* sequence (RefSeq NM000135.2) to amplify the entire coding sequence of *FANCA* mRNA. The six partially overlapping cDNA fragments amplified were 910 bp, 885bp, 980 bp, 910bp, 792 bp and 741bp in size respectively, using 6 pairs of primers (sequences for the same are given in Table 1); covering the 43 exons of *FANCA* gene. The PCR mixture contained (per 25μL) cDNA template 2μL, 1X PCR buffer (10mmol/L Tris-HCl, pH 9.0), 1.5mmol/L MgCl_2_, 0.2 mmol/L deoxynucleoside triphosphate (dNTP), 0.2μmol/L of each primer, and 1.5 units of Taq polymerase (*Genei*, *Bangalore*, *India*). The PCR parameters were optimized as followings: an initial denaturation at 95°C for 5 minutes, 35 cycles of denaturation (95°C for 30 seconds), annealing (51°C—59°C for 1 minute), elongation (72°C for 1 minute), and a final elongation for 7 minutes at 72°C. The PCR amplification products were checked on 2% agarose gel electrophoresis and subsequently sequenced using ABI Prism 3130 Automated DNA Sequencer with forward, reverse primers as well as internal forward and reverse primers designed specifically to cover the entire coding region of *FANCA* gene. The sequences were manually compared with *FANCA* gene RefSeq NM000135.2 using ChromasLite 2.1.1 DNA sequencing software.

**Table 1:** Overlapping Primers for amplifying *FANCA* cDNA.

Primers	Primer Sequence (5’– 3’)	Annealing temperature	Length of product (bp)
FancA1 Forward primer FancA1 Reverse primer	ATCCTGAAAGGGCACAGAAA CAAAGCGTCAAGTGCAAAAA	51°C	910 bp
FancA2 Forward primer FancA2 Reverse primer	TCTGTGCTGCCTTTGTGAAC GTGAGGAGGGAGCGGTACTT	59°C	885 bp
FancA3 Forward primer FancA3 Reverse primer	ACTGGTTCAAGGCCTCCTTT CACACATGGTCCTCACGAAG	57°C	980 bp
FancA4 Forward primer FancA4 Reverse primer	CTGATGGCTGCCTCCAGT TCAGCTACCATCTCCTGCAA	59°C	910 bp
FancA5 Forward primer FancA5 Reverse primer	GGATTTCCACCAAAGCTCAA CTCTCGCAGTCCAGCTTCTT	57°C	792 bp
FancA6 Forward primer FancA6 Reverse primer	AGCTGCTGCACTGCACTTT GCAGGTCCCGTCAGAAGAG	59°C	741 bp

#### Genomic DNA analysis

When a sequence variant was detected in the cDNA amplification products by direct sequencing [8], the corresponding exon with flanking intronic sequence was amplified to confirm the variation at genomic DNA level.

#### *In silico* analysis

PolyPhen-2 (http://genetics.bwh.harvard.edu/pph2/) tool was used to predict possible impact of novel amino acid substitutions on the structure and function of FANCA protein.

## Results

### Chromosome breakage test

The chromosomal breakage analysis of 550 suspected patients at 40ng/mL MMC concentration revealed a high frequency of chromosome breakage (>3 breaks/cell in MMC treated, upto 0.2 breaks/cell in untreated) in 61 patients when compared to appropriate age and sex matched controls (0.1 break/cell in MMC treated, upto 0.03 breaks/cell in untreated).

### FANCD2 monoubiquitination detection by Western blot

**Fig 1:**
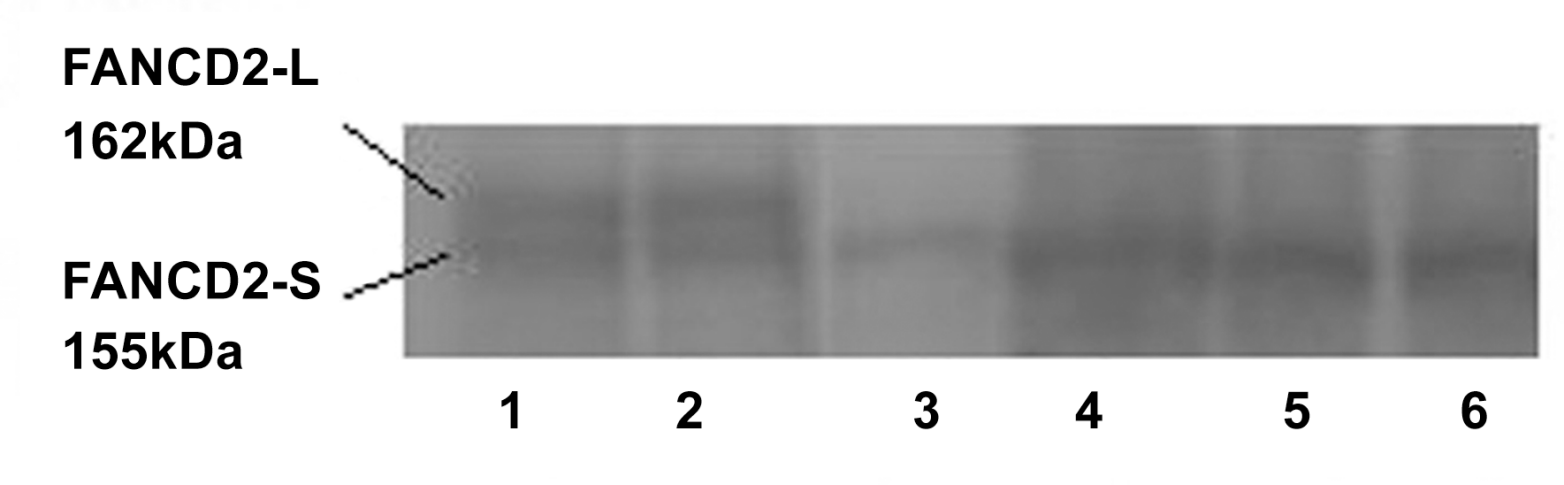
Western blot for FANCD2 monoubiquitination detection. Lane 1 and 2 indicate +MMC and–MMC of negative control (Non FA individual), Lane 3 and 4 indicate +MMC and–MMC of FA (Patient1), and Lane 5 and 6 indicate +MMC and–MMC of FA (Patient2).

All 61 patients were tested positive for FANCD2S+/FANCD2L-Western blot pattern, confirming the diagnosis of FA as well as reaffirming that it was indeed an upstream gene defects in all the Chromosomal breakage positive patients (Figs 1 and S1).

### Mutation analysis

We screened the entire coding sequence of *FANCA* gene in 61 confirmed cases with FA by direct sequencing and MLPA of which 36 patients (34 unrelated individuals and 2 siblings) found to have 26 different *FANCA* molecular changes but only 16 (~26.23%) FA patients (15 unrelated individuals with FA and 1 sibling) found to belong to FA-A complementation group, as other point mutations were heterozygous non-pathogenic or pathogenic mutations and a few were single exon deletions for which exact breakpoints could not be addressed by PCR. The representative results of MLPA and direct sequencing for mutations were presented and classified according to type, frequency, zygosity, consanguinity and occurrence (Tables 2 and 3). In 16 patients, we have identified 15 different molecular changes including 2 (12.5%) missense mutations 6 (37.5%) nonsense mutations, 5 (31.25%) large deletions and 3 (~18.75%) small deletions. In our series 8 novel mutations including 1 small deletion (c.2500delC), 4 non-sense mutations (c.2182C>T, c.2630C>G, c.3677C>G, and c.3189G>A) and 3 missense mutations (c.1273C>G, c.3679G>C, and c.3992 T>C) were identified.

**Table 2:** Deletions detected in FA-A patients correlated with clinical characteristics.

Type of Mutation	Age (yrs)	Sex	Zygosity	Consanguinity in parents	References	Detection Method	Clinical manifestations										
							SS	SP	SkA	B	EyA	EA	OA	CpA	GA	HaP	OthCP
Exon 11 deletion	4	M	Hm	No history of consagnuinity	Novel^a^	MLPA		+	+				+				microcephaly
c.3760_3761delGA	7	F	Hm	Second degree consanguinity	[9,10]	DS		+	+								oncocephaly
Exon 11 deletion	11.5	F	Hm	Third degree consanguinity	Novel*	MLPA	+		+								short neck
Exon 21 deletion	19	F	Hm	Third degree consanguinity	[8]	MLPA	+		+								
Exon 11 deletion	6	F	Hm	Not known	Novel^a^	DS			+			+					
Exon 1–8 deletion	NA	M	Hm	Second degree consanguinity	Fanconi anemia mutation database-Rockfeller University	MLPA	+	+		+							Microcephaly
Exon8-27 deletion	4	M	Hm	Third degree consanguinity	[11]	MLPA			+								multiple minimally enlarged mesenteric lymphnodes
Exon 30 deletion	24	F	Hm	Third degree consanguinity	Fanconi anemia mutation database-Rockfeller University	MLPA		+		+			+				heavy menstrual flow with history of clots, ictericivtirgo
Exon 15–29 deletion	14	M	Hm	No history of consagnuinity	Fanconi anemia mutation database-Rockfeller University	MLPA				+			+	+			tachycardia
Exon1-22 deletion	9	M	Hm	No history of consagnuinity	Fanconi anemia mutation database-Rockfeller University	MLPA	+				+						mongoloid slant
Exon 31 deletion	9	M	Hm	Third degree consanguinity	Fanconi anemia mutation database-Rockfeller University	MLPA		+	+				+				left platynychea
Exon 4–7 deletion	8	M	Hm	Not known	Fanconi anemia mutation database-Rockfeller University	DS	+	+	+		+						
Exon 31 deletion	5	F	Hm	No history of consagnuinity	Fanconi anemia mutation database-Rockfeller University	DS		+	+								low IQ
Exon 6 deletion	9	M	Hm	No history of consagnuinity	Fanconi anemia mutation database-Rockfeller University	DS											Hypoplastic marrow
Exon 11 deletion	16	F	Hm	Not known	Novel^a^	DS	+	+	+							+	
c.3926_3929delCAGA	14	M	Hm	No history of consagnuinity	Fanconi anemia mutation database-Rockfeller University	DS	+	+			+						increased alkaline phosphatase levels
c.2500delC	3	M	Hm	Second degree consanguinity	Novel	DS	+	+			+		+				

SS, Short stature; SP, Skin pigmentation abnormalities; SkA, Skeleton anomalies; B, Bleeding manifestations; EyA, Eye abnormalities; EA, Ear abnormalities; OA, Organ abnormalities; CpA, Cardiopulmonary abnormalities; GA, Genital abnormalities; HaP, High arched palate; OthCP, Other clinical presentations; Hm, Homozygous.

^a^Molecular changes but breakpoint could not be confirmed.

**Table 3:** Single nucleotide substitutions detected in FA patients correlated with clinical characteristics.

Molecular change	Effect on protein	Polyphen-2 score	Age (yrs)	Sex	Zygosity	Consanguinity	References	Detection Method	Clinical Manifestations										
									SS	SP	SkA	B	EyA	EA	OA	CpA	GA	Hap	OthCP
c.4036G>A	p.Ala1346Thr	0.462 (possibly damaging)	10	M	Hz	No history of consanguinity	[12]	DS	+	+	+		+						
c.4036G>A	p.Ala1346Thr	0.462 (possibly damaging)	6	M	Hz	No history of consanguinity	[12]	DS	+		+								
c.3677C>G	p.Ser1226Ter	Early stop gained	14	M	Hm	Not known	Novel	DS				+							High ferritin levels, Echymosis
c.3992T>C	p.Leu1331Pro	1 (probably damaging)	8	M	Hz	No history of consanguinity	Novel	DS	+	+	+		+	+				+	
c.4036G>A	p.Ala1346Thr	0.462 (possibly damaging)	NA	F	Hz	Not known	[12]	DS											Info. Not available
c.1273G>C	p.Asp425His	0.952 (possibly damaging)	13	M	Hz	Not known	Novel	DS		+	+		+		+			+	
c.2574C>G	p.Ser858Arg	0.257(Benign)	7	M	Hz	No history of consanguinity	[13], [14]	DS	+		+	+							suspected AML
c.1303C>T	p.Arg435Cys	1 (probably damaging)	5	F	Hz	Not known	[15], [16], [17]	DS											poor weight gain, loss of weight, loss of appetite, hypoplastic anemia
c.1303C>T	p.Arg435Cys	1 (probably damaging)	9	F	Hz	No history of consanguinity	[15], [16], [17]	DS		+	+				+		+		
c.2630C>G	p.Ser877Ter	Early stop gained	9	F	Hz	No history of consanguinity	Novel	DS		+			+	+	+				
c.2574C>G	p.Ser858Arg	0.257(Benign)	7	F	Hm	No history of consanguinity	[13], [14]	DS		+									Low birth weight
c.163C>T	p.Gln55Ter	Early stop gain	10	F	Hm	Not known	[13],	DS	+	+			+						
c.2574C>G	p.Ser858Arg	0.257(Benign)	5	F	Hm	Second degree consanguinity	[13],[14]	DS		+						+			Dorsalispedis
c.3189G>A	p.Trp1063Ter	Early stop gained	10	F	Hm	No history of consanguinity	Novel	DS		+			+					+	
c.3679G>C	p.Ala1227Pro	1 (probably damaging)	16	M	Hm	No history of consanguinity	Novel	DS	+	+			+						
c.2749C>T	p.Arg917Ter	Early stop gained	11	M	Hm	Second degree consanguinity	Ensembl genome browser	DS		+	+	+							increased levels of LDH, SGPT/OT, necrotic lymphadecitis
c.2851C>T	p.Arg951Trp	1 (probably damaging)	8	M	Hm	Second degree consanguinity	[12], [18–19]	DS		+			+				+	+	microcephaly, Dysmorphic face, alopecia arcate
c.2182C>T	p.Gln728Ter	Early stop gained	9	F	Hm	Second degree consanguinity	Novel	DS	+	+									
c.2182C>T	p.Gln728Ter	Early stop gained	5	F	Hm	Second degree consanguinity	Novel	DS	+										

SS, Short stature; SP, Skin pigmentation abnormalities; SkA, Skeleton anomalies; B, Bleeding manifestations; EyA, Eye abnormalities; EA, Ear abnormalities; OA, Organ abnormalities; CpA, Cardiopulmonary abnormalities; GA, Genital abnormalities; HaP, High arched palate; OthCP, Other clinical presentations; Hm, Homozygous; Hz, Heterozygous.

#### Deletions in *FANCA* gene

The deletions involving exon 6, exon 4–7 were identified in individual patients while four patients showed novel exon 11 deletion and two patients had exon 31 deletion as detected through direct sequencing and MLPA (Table 3). We could also detect 3 different small deletions, c.3760_3761delGA in exon 37, c.3926_3929delCAGA in exon 39 and a novel small deletion c.2500delC in exon 26 by direct sequencing (Table 2). However, we could not confirm the exact cause of single exon deletions in our study by secondary confirmatory method.

#### Single nucleotide substitution mutations in *FANCA* gene

We identified seven different missense mutations in twelve subjects of which three were novel [c.1273C>G (p.Asp425His), c.3679C>G (p.Ala1227Pro), c.3992T>C (p.Leu1331Pro)] and two were recurrent mutations; c.2574C>G in exon 27 and c.4036G>A in exon 41. The heterozygous novel missense variants were analyzed using *in silico* analysis tool *PolyPhen*2 as they were observed at regions of no homology of FANCA protein (Table 4). Of the six different nonsense mutations identified in our study, four were novel [c.2182C>T (p.Gln728Ter), c.2630C>G (p.Ser877Ter), c.3677C>G (p.Ser1226Ter), and c.3189G>A (p.Trp1063Ter)], resulting in early stop gained and formation of truncated protein (Table 3).

**Table 4:** Frequency (%) of malformations exhibited by FA-A patients.

Clinical Presentations	n = 16^a^(%)
Skin Pigmentation (café au lait spots, generalized hyperpigmentation, hypopigmentation)	11(68.75)
Short Stature	9(56.25)
Skeleton anomalies [Thumb (Absent or hypoplastic or supernumerary with thenar eminence) Limb anomalies]	4(25)
Bleeding (epistaxis, other)	4(25)
Microphthalmia, other eye anomalies (hypotelorism)	8(50)
Organ abnormalities or Organomegaly (Renal,spleen,liver)	2(12.5)
Genital abnormality (hypogenitalia, undescended, absent testes, hypoplastic vulva, menstrual problems etc.	1(6.25)
Cardiopulmonary abnormality	1(6.25)

^a^Of 36 cases detected with FANCA molecular changes, only 16 FA cases were assigned FA-A complementation group. Details are explained in the Discussion part.

### Clinical abnormalities

**Fig 2:**
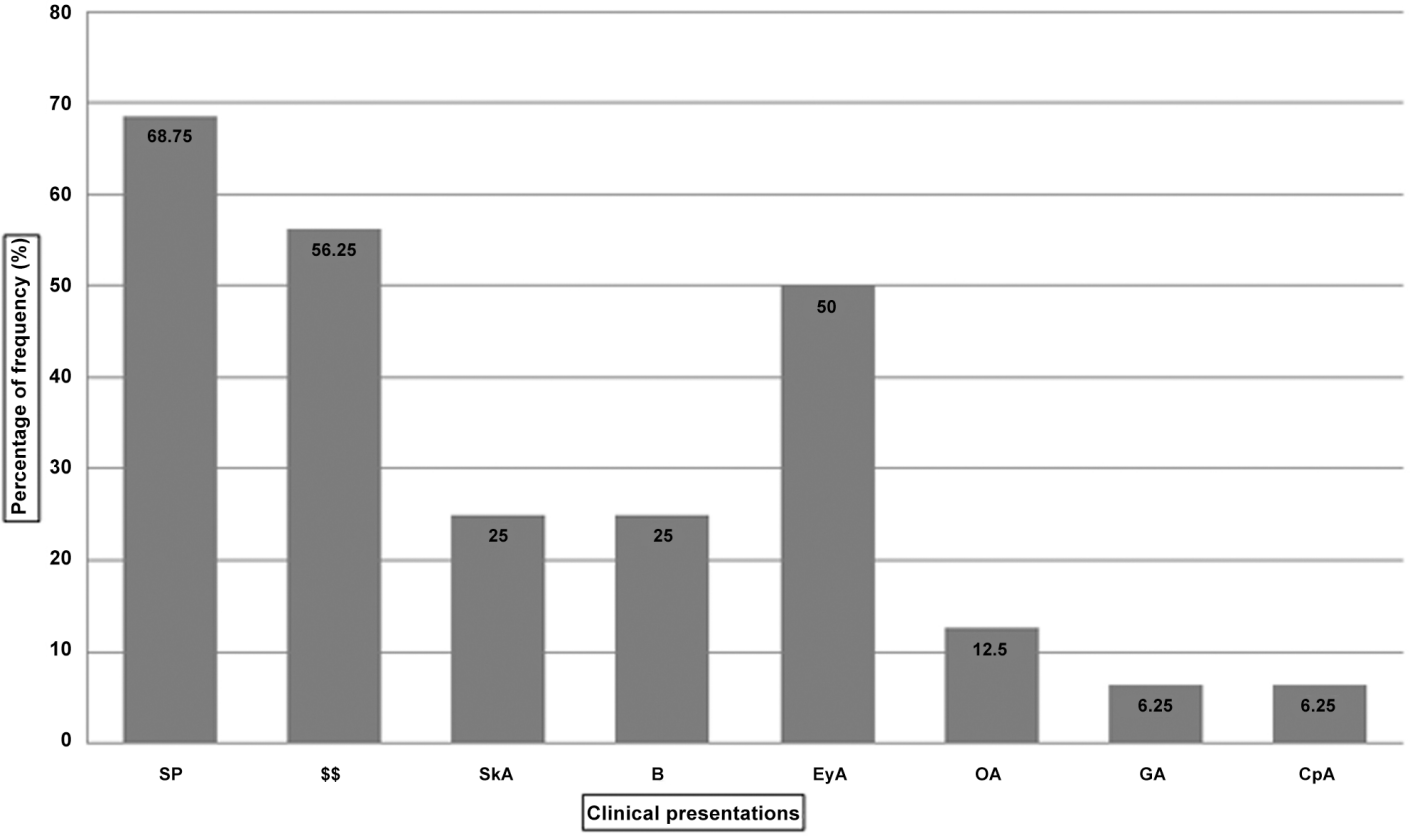
Frequency of clinical abnormalities in FA-A patients. SP, skin pigmentation; SS, short stature; SkA, Skeleton anomalies; B, Bleeding manifestations; EyA, Eye anomalies; OA, Organ abnormalities; GA, Genital abnormality; CpA, Cardiopulmonary abnormalities.

On physical examination, FA specific clinical manifestations were noticed in 16 molecularly confirmed FA-A patients. Apart from bone marrow failure observed in 37.5% patients at the time of diagnosis, other major and minor clinical features of FA were observed at frequencies as described in Fig 1 and Table 4. Skin pigmentation (~68.75%), short stature (~56.25%) and eye anomalies (~50%) were most frequently observed clinical features among FA-A patients whereas, skeleton abnormalities (25%), bleeding manifestations (25%), Organ abnormalities (12.5%), cardiopulmonary abnormalities (6.25%), and genital abnormalities (6.25%) were observed at very low frequency (Fig 2, Table 4).

### Genotype-Phenotype correlation

**Fig 3:**
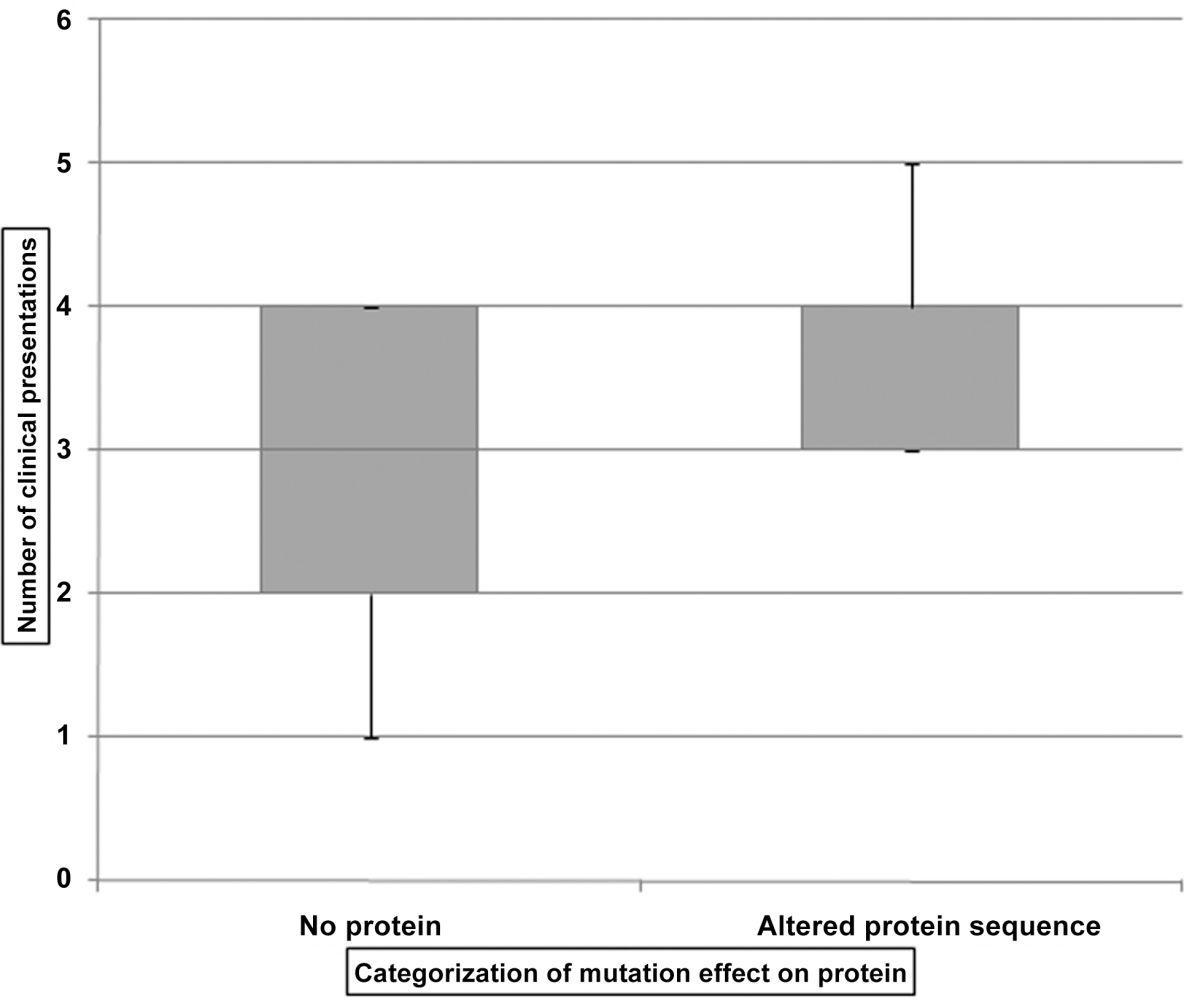
Effect of type of mutation on severity of malformations.

**Fig 4:**
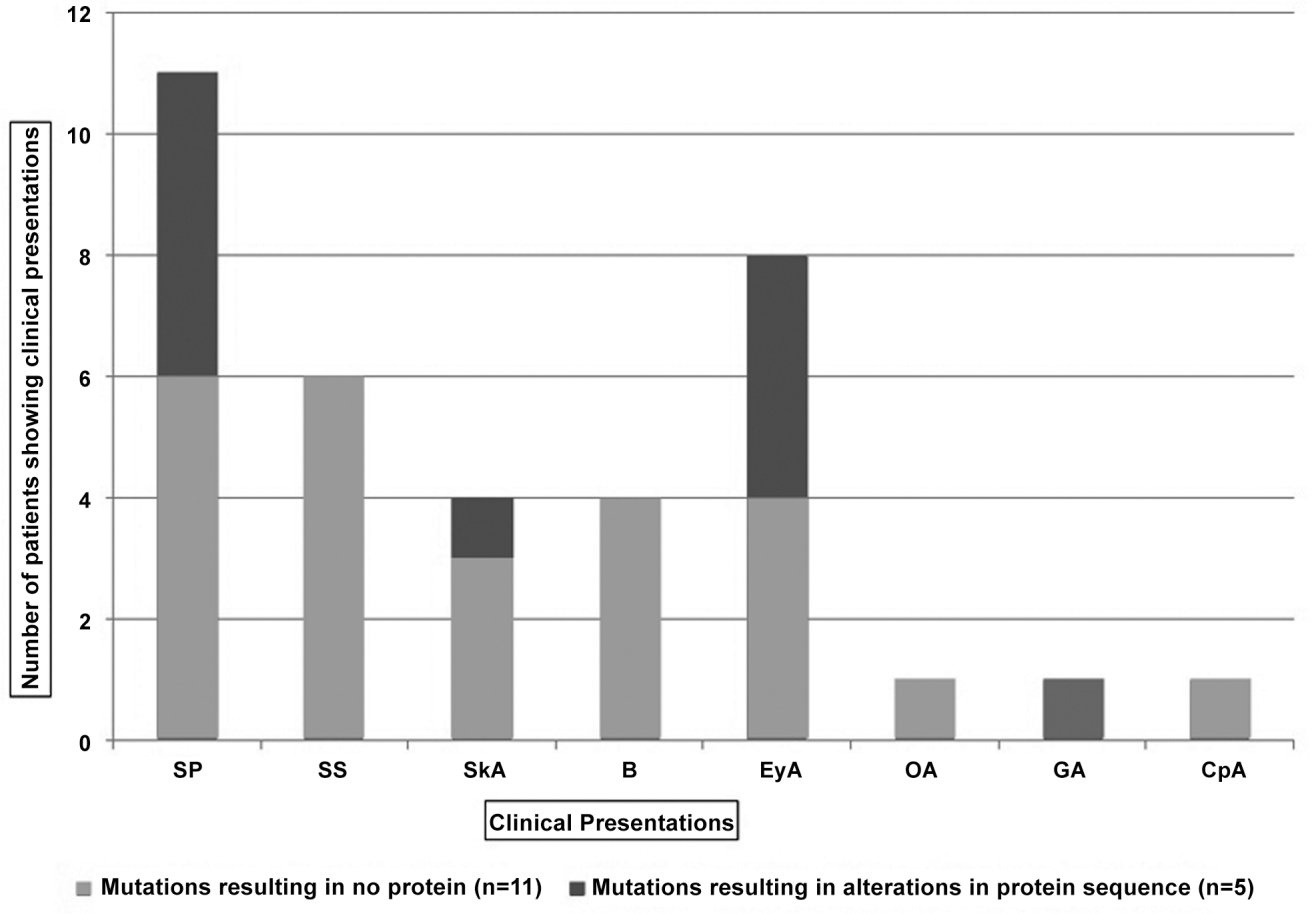
Frequency of clinical presentations observed in genotypes resulting different effects on protein.

The correlation between different types of mutations with severity of phenotype outcomes was studied using a box plot comparing group of patients harboring different types of molecular changes (mutations resulting in no protein and non-sense mutation Vs. Missense mutations and frame shift mutations) of *FANCA* with the number of clinical presentations. It revealed no significant difference in number of malformations among these groups of patients (Fig 3). However, we observed a high frequency of skin pigmentation abnormalities (100%), eye anomalies (80%) in patients harboring *FANCA* nonsense mutations and large deletions resulting in no protein formation compared to those FA-A patients of our study group with missesnse mutations and frameshift mutations resulting in protein alterations (Fig 3 and Table 5). On the other hand, the FA-A patients harboring mutations resulting in protein alterations showed higher frequency of short stature clinical presentation compared to those harboring mutations resulting in no protein formation (Fig 4 and Table 5).

**Table 5:** Frequency of clinical presentations observed in genotypes resulting different effects on protein.

Clinical Presentations	Mutations resulting in no protein formation^a^,n = 11 (%)	Mutations resulting in protein sequence alterations^b^, n = 5 (%)
Skin Pigmentation (café au lait spots, generalized hyperpigmentation, hypopigmentation)	6(54.5%)	5(100%)
Short Stature	6(54.5%)	-
Skeleton anomalies [Thumb (Absent or hypoplastic or supernumerary with thenar eminence) Limb anomalies]	3(27.27%)	1(20%)
Bleeding (epistaxis, other)	4(36.36%)	-
Microphthalmia, other eye anomalies (hypotelorism)	4(36.36%)	4(80%)
Organ abnormalities or Organomegaly (Renal, spleen,liver)	1(9.09%)	-
Genital abnormality (hypogenitalia, undescended, absent testes, hypoplastic vulva, menstrual problems etc.	-	1(20%)
Cardiopulmonary abnormality	1(9.09%)	-

^a^Nonsense mutations and large deletions resulting in no protein formation.

^b^Missense mutations and small deletions resulting in protein sequence alterations.

## Discussion

The physical findings like abnormal skin pigmentation, short stature and abnormalities of thumb, skeleton, eye, kidney and urinary tract are common in FA from birth. Bone marrow failure (BMF) usually develops during first and second decade of life in FA patients. However, FA is suspected only after the onset of pancytopenia in majority of cases. The diagnosis of FA is complicate and a precise diagnosis can be possible with careful clinical and family history data, physical examination and a positive chromosomal breakage investigation. Faivre L et al. reviewed the clinical presentations among two hundred forty five patients from 7 complementation groups (FA-A to FA-G). They could observe high frequency of skin pigmentation abnormalities (74%) in FA-A complementation group patients followed by growth retardation (60%), reduced head circumference (53%) and radial ray defects (49%). In our study though different clinical abnormalities were observed in FA-A patients, the skin pigmentation (68.75%) and short stature (56.25%) were common clinical presentations in FA.

The mutations were common in FA-A, FA-C, and FA-G complementation groups from western population[20]. Several molecular mutations including large deletions, single nucleotide substitutions and small deletion or insertion have been reported in *FANCA* gene[21, 22]. However, no molecular data on FA patients is available from Indian population except for a few reports. Several molecular mutations including large deletions and mutations have been reported for FANCA gene. This is the first study from Indian patients with FA. We have screened FANCA gene mutations by MLPA and direct sequencing in *sixty one* (61) confirmed FA patients with upstream complex gene defect. We screened *FANCA* mutations in 61 confirmed FA cases and we could identify 26 different molecular changes in 36 FA cases (34 unrelated individuals and 2 siblings) including 8 novel mutations (a small deletion c.2500delC, 4 non-sense mutations c.2182C>T, c.2630C>G, c.3677C>G, c.3189G>A; and 3 missense mutations; c.1273G>C, c.3679G>C, and c.3992T>C) (Tables 2 and 3). Among 36 FA cases, 16 cases (15 unrelated individuals and 1 sibling) were assigned FA-A complementation group as these cases found to have homozygous pathogenic molecular changes, and the rest 20 cases either have heterozygous pathogenic mutations or non-pathogenic mutations and a few single exon deletions.

We had used MLPA and cDNA amplification direct sequencing methods for mutation screening. Single exon deletions detected using these techniques, could either be due to variations under probe or primer sequence in the target template in MLPA or PCR for genomic DNA or due to splice site donor or acceptor site mutations while using cDNA as template for amplification. Therefore, we have not considered *FANCA* single exon deletions (9/17, 52.94%) found in confirmed FA cases for assigning FA-A as their complementation group. Nine of heterozygous mutations were detected in our study group, of which four (4/9; 44.4%) are with no significant disease pathogenesis effect and five (5/9; 55.6%) although with pathogenic potential could not be taken in to consideration for assigning complementation group due to heterozygosity and non-existence of biallelic mutations. Due to existence of Nonsense Mediated Decay (NMD) mechanism[23], transcripts with non-sense mutations are degraded before getting translated into proteins. These heterozygous non-sense mutations might have not been picked up while screening cDNA for *FANCA* molecular changes and thus frequency (16/61; 26.23%) of FA-A complementation group is found to be less in our study group compared to that observed world-wide.

**Fig 5:**
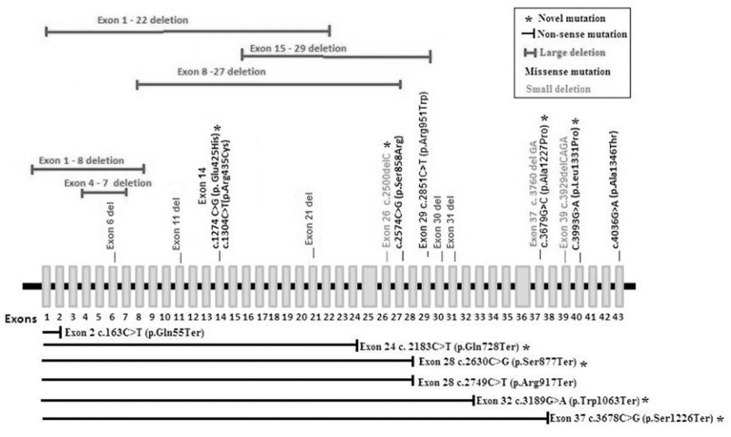
Exon-wise mutation spectrum in FANCA gene with random distribution of mutations throughout FANCA gene and large deletions are present till exon 31. Novel mutations are indicated by asterisk mark (*), large deletions in red, small deletions in green, 22 nonsense mutations in black with each nonsense mutation indicated by horizontal line ending with vertical line at the exon where the mutation results in early stop gained for the transcript, and the missense mutations are mentioned above respective exons in black.

*FANCA* is a large gene spanning 43 exons, harboring varied molecular changes including single nucleotide substitutions, deletions and insertions leading to altered protein structure and function. The large intragenic deletions in *FANCA* have been reported as result of Alu-Alu mediated recombination in previous studies reported by Morgan et al., 1999 and Tipping et al., 2001, and Flynn EK et al., 2014 [21, 24–25]. Large deletions and single exon deletions detected in our study group could be due to their positions relative to Alu sequence repeats (Table 6). The majority of FA-A patients in our study harbored large deletions (5/16; 31.25%), nonsense mutations (6/16; 37.5%) followed by small deletions (3/16; 18.75%) and missense mutations (2/16; 12.5%). Our study highlights random distribution of mutations throughout *FANCA* gene, interestingly however, large deletions were present till exon 31 and same was discussed by Shukla et al., 2013 (Fig 5).

**Table 6:** Number of Alu repeats and their positions relative to large deletions found in FA-A patients.

Large Deletions	Exon 1–8 deletion	Exon 4–7 deletion	Exon 6 deletion	Exon 8–27 deletion	Exon 11 deletion	Exon 21 deletion	Exon 30 deletion	Exon 31 deletion	Exon 1–22 deletion	Exon 15–29 deletion
Number of Alu repeats; their relative position relative to deletions	16, 7; 5’-UTR region, intron 9	3,3; Intron 3, intron 7	3;Intron 6	3,2;Intron 7, intron 27	a	3; Intron 21	4;Intron 30	a	16, 1;5’-UTR region, intron 22	3,6;Intron14, intron 29
Reference	[21]	[21]	[21]	[21]	[24]	[21]	[21]	[24]	[21]	[21]

^a^ As reported by Tipping et al., 2001, a high degree of homology has been shared by a region of intron 11 and intron 31 with several subfamilies of *Alu* repeat sequence.

Few studies have reported the genotype–phenotype correlations in FA [3, 5, 26]. However significant correlation was not observed between the mutations and phenotype of FA patients. In our study we have correlated the mutations with FA phenotype. Large intragenic deletions and nonsense mutations result in no protein formation, and thus are grouped together while comparing genotype-phenotype correlation against frame-shift mutations (small deletions) and missense mutations causing protein sequence alterations. Polyphen-2 tool was used to predict the effect of mutation or variations on FANCA protein structure and function. We have therefore excluded these benign heterozygous and homozygous variations from FA-A complementation group and genotype-phenotype correlation study. We could not find any significant differences in severity of phenotype between two genotype categories (mutations resulting in no protein formation versus those causing protein sequence alterations). We could mark existence of a high frequency of skin pigmentation abnormalities and eye anomalies in patients harboring *FANCA* molecular changes resulting in no protein formation compared to those FA-A patients in our study group with mutations resulting in protein alterations. But the latter group of FA patients showed higher frequency of short stature clinical presentation compared to those harboring mutations resulting in no protein formation.

FA being a recessive genetic disorder, parental consanguinity is an important factor to determine the underlying genetic cause for mutational changes in FA patients, for prenatal diagnosis and genetic counseling of FA families. Of the 36 cases screened for mutational analysis, parental consanguinity was consistent with 19 cases (~52.8%), consanguinity was not known in 8 cases (22.2%), while 9 cases (25%) showed discrepancy between the zygosity of mutation and parental consanguinity. As we have observed ~52.8% zygosity of mutations in FA patients consistent with consanguinity of parents, the underlying cause could be inheritance of the carrier mutation from parent(s). The discrepancies were seen only in homozygous molecular changes including 3 single exon deletions (a Exon 11 deletion, a Exon 6 deletion, and a Exon 31 deletion), 3 single nucleotide substitutions (c.2574C>G, c.3189G>A, and c.3679G>C), two large deletions (Exon 15–29 deletion and Exon1-22 deletion), and a small deletion case (c.3926_3929delCAGA) (Tables 2 and 3). As *FANCA* is a large gene with heterogenous spectrum of mutations, absence of biallelic mutations and the fact that some mutations may be intronic or regulatory also makes the completion of many mutation screening in FA cases difficult. An effective molecular screening strategy should therefore be developed for confirmation of these mutations and determining the breakpoints for single exon deletions. Determination of family pathogenic variants is the ultimate confirmation of diagnosis and is necessary for genetic counseling and management of the disease.

## Supporting Information

S1 Fig Western blot for detection of FANCD2 monoubiquitination in 61 patients screened for upstream or downstream complex gene defect. ‘+’ indicates monoubiquitination event occurring through DNA damage induced by MMC and ‘-’ indicates spontaneous monoubiquitination.
(TIF)

## Abbreviations

FA: Fanconi anemia
MMC: MitomycinC
DEB: Diepoxybutane
PHA: Phytohemagglutinin
MLPA: Multiplex Ligation-dependent Probe Amplification
NLS: Nuclear localization sequence
ORF: open reading frame

## References

[1] D'Andrea AD, Grompe M. 2003 The Fanconi anaemia/BRCA pathway. Nat Rev Cancer.3:23–34. 12509764 10.1038/nrc970

[2] Meetei AR, Levitus M, Xue Y, Medhurst AL, Zwaan M, Ling C, et al 2004 X-linked inheritance of Fanconi anemia complementation group B. Nat Genet. 36(11):1219–1224. 15502827 10.1038/ng1458

[3] Faivre L, Guardiola P, Lewis C, Dokal I, Ebell W, Zatterale A, et al 2000 Association of complementation group and mutation type with clinical outcome in Fanconi anemia. Blood. 96: 4064–4070. 11110674

[4] Bogliolo M, Surralles J. 2015 Fanconi anemia: a model disease for studies on human genetics and advanced therapeutics. Curr Opin Genet Dev. 33: 32–40. 10.1016/j.gde.2015.07.002 26254775

[5] Neveling K, Endt D, Hoehn H, Schindler D. 2009 Genotype-phenotype correlations in Fanconi anemia. Mut Res. 668:73–91. 19464302 10.1016/j.mrfmmm.2009.05.006

[6] Sasaki MS, Tonomura A. 1973 A high susceptibility of Fanconi’s anemia to chromosome breakage by DNA cross-linking agents. Cancer Res. 33:1829–1836. 4352739

[7] Oostra AB, Nieuwint AWM, Joenje H, Winter JP. 2012 Diagnosis of Fanconi Anemia: Chromosomal Breakage Analysis. Anemia. 2012: 1–9. 10.1155/2012/238731 PMC3368163 22693659

[8] Gille JJP, Floor K, Kerkhoven L, Ameziane N, Joenje H, Winter JP. 2012 Diagnosis of Fanconi Anemia: Mutation Analysis by Multiplex Ligation-Dependent Probe Amplification and PCR-Based Sanger Sequencing. Anemia. (34): 1–13. 10.1155/2012/603253 PMC3388349 22778927

[9] Solomon PJ, Margaret P, Rajendran R, Ramalingam R, Menezes GA, Shirley AS, et al 2015 A case report and literature review of Fanconi Anemia (FA) diagnosed by genetic testing. Ital J Pediatr. 41(38): 1–8. 25953249 10.1186/s13052-015-0142-6 PMC4438458

[10] Arthur NBJ, Ganapule AP, Palani D, Viswabandya A, Mathews V, Abraham A, et al. 2014. Clinical and Molecular Characterization of Fanconi Anemia: An Indian Perspective. 56th ASH Annual Meeting and Exposition, Session: 508.

[11] Shukla P, Rao A, Ghosh K, Vundinti BR. 2013 Identification of a novel large intragenic deletion in a family with Fanconi anemia: First molecular report from India and review of literature. Gene. 518: 470–475. 10.1016/j.gene.2013.01.016 23370339

[12] Ameziane N, Errami A, Leveille F, Fontaine C, de Vries Y, van Spaendonk RM, et al 2008 Genetic subtyping of Fanconi anemia by comprehensive mutation screening. Hum Mutat. 29(1):159–166. 17924555 10.1002/humu.20625

[13] Wijker M, Morgan NV, Herterich S, van Berkel CG, Tipping AJ, Gross HJ, et al 1999 Heterogeneous spectrum of mutations in the Fanconi anaemia group A gene. Eur J Hum Genet.7(1):52–59. 10094191 10.1038/sj.ejhg.5200248

[14] Tamary H, Bar-Yam R, Shalmon L, Rachavi G, Krostichevsky M, Elhasid R, et al 2000 Fanconi anaemia group A (FANCA) mutations in Israeli non-Ashkenazi Jewish patients. Br J Haematol. 111(1):338–343. 11091222 10.1046/j.1365-2141.2000.02323.x

[15] Tachibana A, Kato T, Ejima Y, Yamada T, Shimizu T, Yang L, et al 1999 The FANCA gene in Japanese Fanconi anemia: reports of eight novel mutations and analysis of sequence variability. Hum Mutat.13(3):237–44. 10090479 10.1002/(SICI)1098-1004(1999)13:3<237::AID-HUMU8>3.0.CO;2-F

[16] Adachi D, Oda T, Yagasaki H, Nakasato K, Taniguchi T, D'Andrea AD, et al 2002 Heterogeneous activation of the Fanconi anemia pathway by patient-derived FANCA mutants. Hum Mol Genet. 25:3125–34. 10.1093/hmg/11.25.3125 12444097

[17] Yagasaki H, Hamanoue S, Oda T, Nakahata T, Asano S, Yamashita T. 2004 Identification and characterization of novel mutations of the major Fanconi anemia gene FANCA in the Japanese population. Hum Mutat. 24(6):481–490. 15523645 10.1002/humu.20099

[18] Chandra S, Levran O, Jurickova I, Maas C, Kapur R, Schindler D, et al 2005 A rapid method for retrovirus-mediated identification of complementation groups in Fanconi anemia patients. Mol Ther. 12(5):976–984. 16084127 10.1016/j.ymthe.2005.04.021

[19] Levran O, Diotti R, Pujara K, Batish SD, Hanenberg H, Auerbach AD. 2005 Spectrum of sequence variations in the FANCA gene: an International Fanconi Anemia Registry (IFAR) study. Human Mutation. 25(2): 142–149. 15643609 10.1002/humu.20125

[20] Shimamura A, Alter BP. Pathophysiology and management of inherited bone marrow failure syndromes. Blood Rev 2010: 24: 101–122. 10.1016/j.blre.2010.03.002 20417588 PMC3733544

[21] Morgan NV, Tipping AJ, Joenje H, Mathew CG. High frequency of large intragenic deletions in the Fanconi anemia group A gene. Am J Hum Genet 1999 (65): 1330–1341. 10.1086/302627 PMC1288285 10521298

[22] Moghrabi NN, Johnson MA, Yoshitomi MJ, Zhu X, Al-Dhalimy MJ, Olson SB, et al Validation of Fanconi anemia complementation Group A assignment using molecular analysis. Genet Med 2009: 11: 183–192. 10.1097/GIM.0b013e318193ba67 19367192

[23] He F, Jacobson A. Nonsense-Mediated Decay: Degradation of Defective Trancript is only part of the story. Annu Rev Genet 2015(49):339–366. 26436458 10.1146/annurev-genet-112414-054639 PMC4837945

[24] Tipping AJ, Pearson T, Morgan NV, Gibson RA, Kuyt LP, Havenga C, et al 2001 Molecular and genealogical evidence for a founder effect in Fanconi anemia families of the Afrikaner population of South Africa. Pro of the Natl Acad of Sci of USA. 98(10): 5734–5739. 10.1073/pnas.091402398 PMC33282 11344308

[25] Flynn EK, Kamat A, Lach FP, Donovan FX, Kimble DC, Narisu N, et al 2014 Comprehensive analysis of pathogenic deletion variants in Fanconi anemia. Human Mutation. 35(11): 1342–1353. 10.1002/humu.22680 25168418 PMC4407816

[26] Castella M, Pujol R, Callén E, Trujillo JP, Casado JA, Gille H, et al 2011 Origin, functional role, and clinical impact of Fanconi anemia *FANCA* mutations. Blood. 117(14):3759–69. 10.1182/blood-2010-08-299917 21273304 PMC3083295

