# Supplementary figures and images for: FANCA Gene Mutations with 8 Novel Molecular Changes in Indian Fanconi Anemia Patients
Source: PLoS One. 2016 Jan 22;11(1):e0147016. doi: 10.1371/journal.pone.0147016 (PMC4723128; doi:10.1371/journal.pone.0147016)

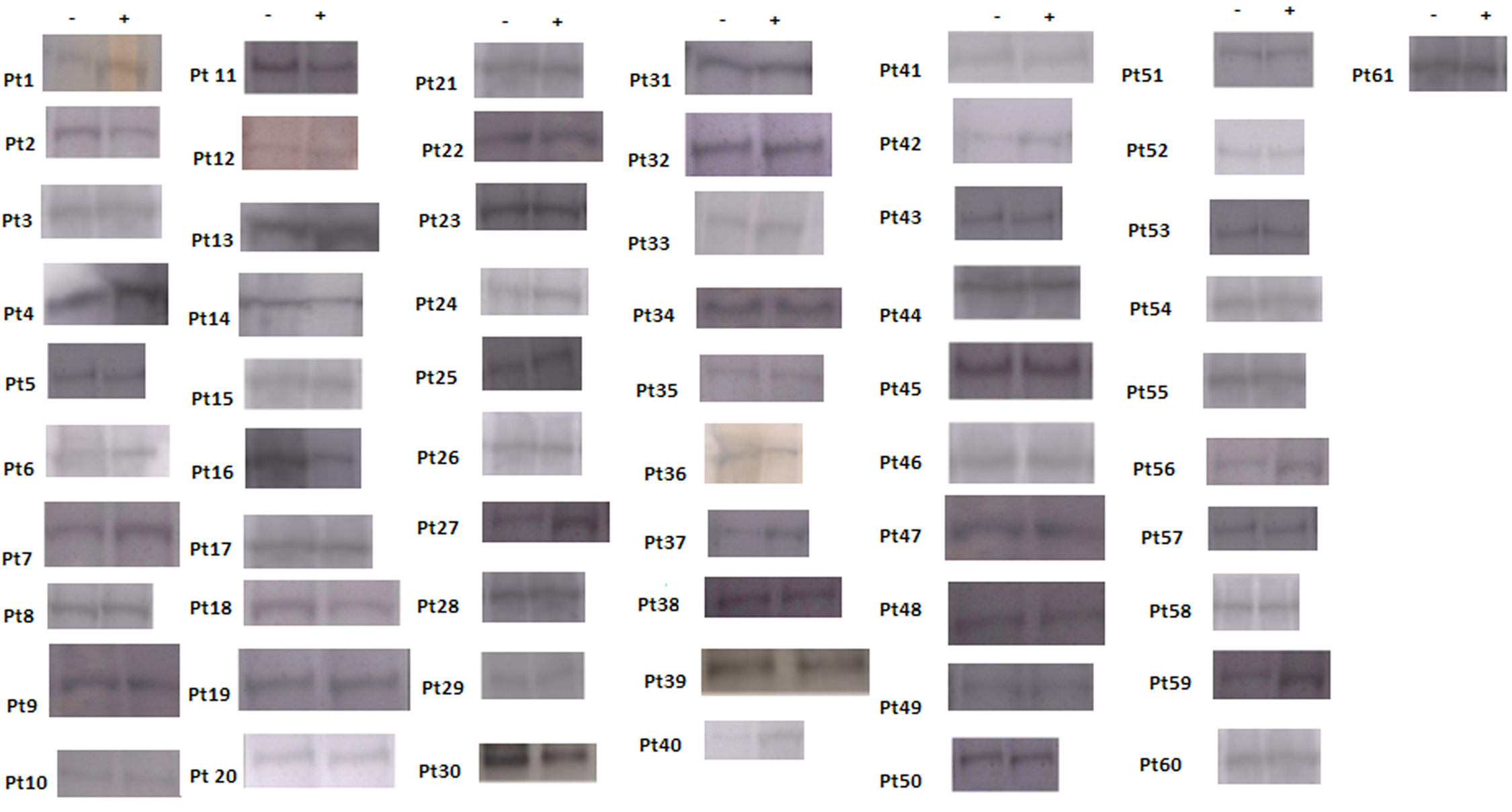

Supplement: S1 Fig — ‘+’ indicates monoubiquitination event occurring through DNA damage induced by MMC and ‘-’ indicates spontaneous monoubiquitination. (TIF) [file pone.0147016.s001.TIF]
